# Supplementary material for: Diethylcarbamazine elicits Ca2+ signals through TRP-2 channels that are potentiated by emodepside in Brugia malayi muscles
Source: Antimicrob Agents Chemother. 2023 Sep 20;67(10):e00419-23. doi: 10.1128/aac.00419-23 (PMC10583680; doi:10.1128/aac.00419-23)
Supplement: Fig. S1 Legend — Legend for Fig. S1. [file aac.00419-23-s0001.docx]

**Supplementary Figure 1: Combination of all 30 µM diethylcarbamazine and 10 mM CaCl_2_ recordings in untreated and dsRNA *trp-2­* treated muscles:** A) Combination of all recordings in response to 30 µM DEC in untreated *Brugia* muscles. Red trace indicates sample presented in figure 5. Light brown box highlights diethylcarbamazine application for 5 minutes. B) Combination of all 10 mM CaCl_2_ in untreated *Brugia* muscles. Red trace indicates sample presented in Figure 5. Grey box indicates 10 mM CaCl2 application. *n* = 5 muscles from 5 individual *Brugia* diethylcarbamazine treatments and corresponding 10 mM CaCl_2_. C) Combination of all dsRNA *trp-2* treated muscles to 30 µM DEC. Red trace indicates sample presented in Figure 5. Light brown box highlights diethylcarbamazine application for 5 minutes. D) Combination of all 10 mM CaCl_2_ in dsRNA *trp-2* treated *Brugia* muscles. Red trace indicates sample presented in Figure 5. Grey box indicates 10 mM CaCl2 application. *n* = 3 muscles from 3 individual *Brugia* for dsRNA *trp-2* diethylcarbamazine treatments and corresponding 10 mM CaCl_2_.
