# Supplementary figures and images for: Diethylcarbamazine elicits Ca2+ signals through TRP-2 channels that are potentiated by emodepside in Brugia malayi muscles
Source: Antimicrob Agents Chemother. 2023 Sep 20;67(10):e00419-23. doi: 10.1128/aac.00419-23 (PMC10583680; doi:10.1128/aac.00419-23)

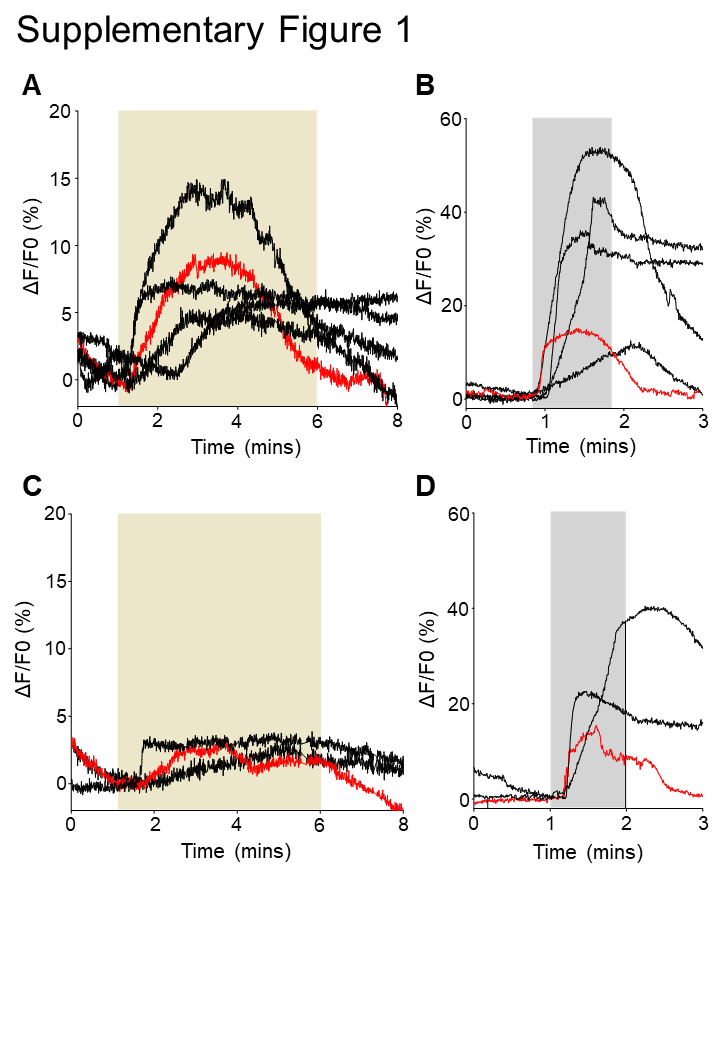

Supplement: Fig. S1 — Effect of RNAi of TRP-2 inhibiting DEC responses. [file aac.00419-23-s0002.tif]
