# Supplementary material for: Diethylcarbamazine elicits Ca2+ signals through TRP-2 channels that are potentiated by emodepside in Brugia malayi muscles
Source: Antimicrob Agents Chemother. 2023 Sep 20;67(10):e00419-23. doi: 10.1128/aac.00419-23 (PMC10583680; doi:10.1128/aac.00419-23)
Supplement: Tables S1 AND S2 — Table S1: List of Primers used in the study Table S2: dsRNA probe sequences. [file aac.00419-23-s0003.docx]

**Supplementary Table 1: List of primers used in this study.**

| **Primer Name** | **Description** | **Sequence (5’ – 3’)** |
| --- | --- | --- |
| *trp-2f2* | *Bma trp-2 5’* | AAGAAGTACGTGGACCACCA |
| *trp-2r2* | *Bma trp-2 3’* | TCGAAGTGCAACGGTACATA |
| *trp-2fT7* | *Bma trp-2 dsRNA PCR with t7 promoter 5′* | TAATACGACTCACTATA AAGAAGTACGTGGACCACCA |
| *trp-2rT7* | *Bma trp-2 dsRNA PCR with t7 promoter 3′* | TAATACGACTCACTATA TCGAAGTGCAACGGTACATA |
| SSK 5F | *Bma GAPDH 5’* | GACGCTTCAAGGGAAGTGTTTCTG |
| SSK 5R | *Bma GAPDH 3’* | GTTTTGGCCAGCACCACGAC |
| LacZf | *LacZF dsRNA 5’* | CGTAATCATGGTCATAGCTGTTTC |
| LacZr | *LacZR dsRNA 3’* | CTTTTGCTGGCCTTTTGCTC |
| LacZft7 | *LacZ dsRNA with t7 promoter 5’* | TAATACGACTCACTATAGGGCGTAATCATGGTCATAGCT GTTTC |
| LacZrt7 | *LacZ dsRNA with t7 promoter 3’* | TAATACGACTCACTATAGGGCTTTTGCTGGCCTTTTGCTC |

**Supplementary Table 2: dsRNA probe sequences**

| dsRNA Probe | Sequence 5’ – 3’ |
| --- | --- |
| *trp-2* | AGAAGTACGTGGACCACCACCAACACTCGTTGAATTAGCTATTCTAACATGGGTTTTTGGTTTGGTATGGGTTGAAATTAAGCAATTATGGAATGAAGGCCTTTGTGATTATTGTTCAGATTTGTGGAATATTCTTGACTTCATAACTAATGCATTGTATTTATGTACCGTTGCACTTCGA |
| *LacZ* | CGTAATCATGGTCATAGCTGTTTCGGTTACGATGCGCCCATCTACACCAACGTGACCTATCCCATTACGGTCAATCCGCCGTTTGTTCCCACGGAGAATCCGACGGGTTGTTACTCGCTCACATTTAATGTTGATGAAAGCTGGCTACAGGAAGGCCAGACGCGAAGAGCAAAAGGCCAGCAAAAG |
